# Methylene Blue Based Device for Pathogen Reduction in Human Plasma

**Published:** 2013-07-22

**Authors:** A Elikaei, S M Hosseini, Z Sharifi, H Latifi, H Nikbakht, H Mirshafiee, A Asadollahi

**Affiliations:** 1 Virology Laboratory, Research Centre of Iranian Blood Transfusion Organization (IBTO), Tehran, Iran.; 2 Department of Microbiology, Faculty of Biological Science Shahid Beheshti University, GC, Tehran, Iran.; 3 Laser and Plasma Research Institute, Shahid Beheshti University, GC, Tehran, Iran.

**Keywords:** Blood-borne Pathogens, Plasma, Methylene Blue

## Abstract

**Background:**

Despite improvement in safety of plasma transfusion some virus transmission still remains a problem. So as World Health Organization (WHO) recommends, many countries developed Pathogen Reduction Technologies (PRT) to inactivate pathogens, in plasma components. The Methylene Blue (MB) based methods is one of the most universal one. The purpose of this research was, produce a device that can inactivate viruses in MB environment.

**Materials and Methods:**

In this interventional study, each Plasma Sample was illuminated by 70Pieces (PCs) of 1 w red Light Emitting Diodes (LEDs) from one side. These LEDs emit light at central wavelength of 627 nm with 20 nm Full Width at Half Maximum (FWHM). Two model viruses Herpes Simplex Virus (HSV) and Vesicular Stomatitis Virus (VSV) were used and Tissue Culture 50% Infection Dose (TCID_50_) was used to calculate virus Log reduction. Two concentration of MB and 5 different illumination times were used.

**Results:**

In 10 µm concentration of MB, HSV had 6.00±0.2 maximum log reduction that obtain after 60 minutes illumination and VSV had 5.50± 0.3 maximum log reduction after 75 minutes illumination. In 1 µM concentration of MB, HSV had 5.20±0.3 maximum log reduction that obtain after 60 minutes illumination and VSV had 4.90± 0.2 maximum log reduction after 75 minutes illumination.

**Conclusion:**

Results of virus inactivation in this method were similar to other methods (P-value<0.05 in comparison with Spring method, and P-value>0.05 in comparison with Theraflex), and it showed this device could inactivate viruses according to WHO recommendation.

## Introduction

Human plasma is a source of important products which are obtained by a combination of large-scale whole blood processing steps (1). So its safety is critical in its transfusion and fractionation. Despite improvements in safety of plasma transfusion due to selection of the donors, performance of serological screening and Nucleic Acid Testing (NAT), break through infection still occur (2). The risks of viral infection are due to the window period, emerging pathogens and false negative or technical errors (3-6). So in certain countries, the safety enhances using Pathogen Reduction Technologies (PRTs). More recently, several PRTs have been developed to allow treatment of blood products (7). The major pathogen reduction methods currently available for plasma involve the use of Solvent Detergent (SD), Methylene Blue (MB), Amatosalen and riboflavin as additives. MB treatment involves exposure to visible light, and the last two methods are used in combination with ultra violet light (8). Each method has its advantage and disadvantage. Effectiveness against pathogens is the critical point to use of these procedures when making comparisons, however, it is important that pathogen reduction results be reported using conditions that represent the commercially reduction factor (9). 

Phenothiazine based photosensitizers have been employed in photo antimicrobial research for nearly 80 years, both as pioneer and novel compounds (10). Methylene Blue is a phenothiazine dye which its treatment in plasma has been used in clinical use for 15 years (11). When activated by visible light, MB generates reactive oxygen free radicals, mainly singlet oxygen, through a type II photodynamic reaction and these are responsible for its pathogen inactivation properties (12).

The first method for using MB in PRT developed by Mohr and Lambrecht (13), however, that method is not marketed by commercial companies.

MB pathogen inactivation technology is a photodynamic procedure using MB and visible light and applied to single donor units of plasma. After addition of MB to the plasma, it intercalates into nucleic acids and after illumination by visible light (630 nm wave lengths, 180 joules/cm^2^), singlet oxygens are formed and destroy the viral nucleic acids (14-16).

There are several fundamental differences in the process for two important commercial MB pathogen inactivation methods, (the Theraflex system compared to the Spring method). The time for illumination and its source (and energy dose) in these two methods are different. Based on available data, all methods demonstrate effectiveness against common pathogen including HIV (8). So, this method can be used as a PRT system in many countries. 

The purpose of this research was designation and produces a device using red LED lamps to inactivate model viruses in human plasma in combination with MB.

## Materials and Methods

**Device designation and production**

The bag was illuminated by 70 Pieces (PCs) of 1 w (nominal power dissipation) red Light Emitting Diodes (LEDs) from one side. These LEDs emit light at central wavelength of 627 nm with 20 nm FWHM. The distance from the center of the bag to LED array was 4.5 cm. Light intensity profile simulation and optimization was made with considering intensity profile of each LED and location of LEDs in the array. Location of LEDs was optimized to maximize the illuminated power on the bag and maximize intensity uniformity, simultaneously. After optimization, 75 percent of radiated power reached the bag and intensity difference in the bag was below 20 percent of the maximum intensity. To prevent LEDs failure they were cooled by heat sink and fan, also the bag was cooled down with another fan to prevent increase in plasma temperature for maintaining plasma quality. The device has a control circuit to control the time of illumination and temperature of LEDs, and it has been programmed to ensure that the temperature of LEDs never reach 40 ^°^C. During all illumination process temperature of bags was kept 26± 2^°^C.

**Preparation and qualification of model viruses**

Viruses that were evaluated included two enveloped viruses, Herpes simplex Virus (HSV; *Herpesviridae, Alpha herpes virinae, Herpes Simplex Virus, *Type 1) and Vesicular Stomatitis Virus (VSV; *Rhabdoviridae, Vesiculovirus, Indian strain*) models for Hepatitis B virus. Model viruses obtained from virology laboratory of IBTO (Iranian Blood Transfusion Organazation) (17). 

Fifty percent Tissue Culture Infection Dose (TCID_50_) was used for virus titration. The Vero cells (NCBI:C101) were grown in Dulbecco's Modified Eagle's Medium (DMEM) (Gibco, U.K.) supplemented with 10% fetal calf serum (Gibco, U.K.) at 37°C in a humid atmosphere of 5% CO_2_. Antibiotics of penicillin and streptomycin (Gibco, U.K.) were added in 1% concentration to the cell culture media. Confluent monolayer of cells were grown in T25 flasks (NUNC, Denmark) and infected with 0.5 ml of the appropriate virus suspension. Once complete Cytopathic Effect (CPE) of the monolayer had occurred, they were subjected to rapid freezing and thawing and then following by low-speed centrifugation (10 min at 1200g) to sediment cell debris. Viruses were frozen at -80°C for assessment of tissue culture infectivity dose at 50 % (17).

Vero cell line cultured in sterile 96 well cell culture plates (NUNC, Denmark), and after infection with model viruses, the TCID_50_ was calculated based on the method of Spearman and Karber and expressed as log TCID_50_(18).

**Pathogen inactivation process**

MB for intravenous application (Merck) was used. Two MB concentration applied for this study. Each ampoule was formed 1ml solution of 1% and 10 % dye. The final concentrations of MB in plasma sample were1 and 10 µmol /L (16). Whole blood donations (500 ml in 70 ml CPD) to obtain from voluntary unpaid blood donors screened for Hepatitis C Virus (HCV), Hepatitis B Virus (HBV) and Human Immunodeficiency Virus (HIV). Then, using a filter leuko reduction was performed. 50 ml of plasma sample added to a new universal bag (Baxter) and MB and model viruses spiked.

One ml of each prepared virus added to 10 ml of plasma sample. The virus-containing plasma was pre-incubated in dark for 1h at 4^°^c (17). The light emitting was used from one side for 30, 45, 60 and 75 minutes. Before illumination and pathogen inactivation, the device was controlled for Light dose, LED illumination and temperature.

**Infectivity assays**

The TCID_50_ was calculated based on the method of Spearman and Karber and expressed as log TCID_50_ (18, 19). 

Each assay and illumination in this process has repeated 3 times (n= 3). The overall reduction factor was expressed as the sum of each stapes's log reduction factor. The reduction factors were calculated using the equation: 

R= log A_ °_ - log A_n_

Where R is the reduction factor, A_°_ is the total virus load after spiking, and A_n_is the total virus load in the treated sample (20). Viral titers were determined by endpoint titration in micro titer plate assays. (1:8 serial dilution, eight parallel samples were used).

The limit of detection of the infectivity assay is the lowest level of virus that statistically can be detected. It is dependent on the volume and dilution of the test item applied to the assay. When the virus titers reached the limit of detection of the standard titration, large- volume plating was performed to decrease the limit of detection of the assay (20).

**Controls**

Several different controls were used to compare with test samples. Negative controls included: plasma without virus spiking, and supernatants of cell line that no virus spiked in them. Positive controls were plasma plus virus without any treatment and cell line with virus spiking. MB without illumination and light without MB were used to measure each factor solely. and DMEM which were used to dilute MB and prepare viruses, respectively were used to find their impact on pathogen reduction process. 

**Statistical analysis**

SPSS 16 was used for data analyzes. P-value<0.05 was considered statistically significant.

## Results

All cell cultures in 96 well flat-bottom plates were studied microscopically for presence of viral infection. Quantitative infectivity assay were performed according to one of the end points titration methods (17). Each well was scored simply as positive or negative, and the results were converted to a titer (log median TCID50), using Spearman and Karber (18, 19). Cell changes in infected samples were compared with the related untreated cell lines. In positive controls virus titrations were near to virus load and considered as virus titrations after 0 minutes illumination, and both negative controls had no CPE. The effect of MB treatment depends on the combined action of the photoactive dye and light (Table I). Illumination in the absence of dye for 1 h could not decrease virus titration significantly, as well as incubation in the presence of MB in both concentrations in dark for 1h. Phosphate Buffer Saline(PBS) and DMEM had no effects on viruses’ titers in plasma. Plasma with HSV was more sensitive to this pathogen reduction system than VSV, but their difference was not significant (P-value> 0.05).

Different illumination times resulted different energy doses, and increasing in light doses increased log reduction significantly (P-value< 0.05). Five different times were used: 0, 30, 45, 60 and 75 minutes that results 5different light doses that were: 0, 27, 40, 54, and 70 J/cm2. In 10 µm concentration of MB, HSV had6.00± 0.2 maximum log reduction that obtained in 54 J/cm2 after 60 minutes illumination and VSV had 5.30± 0.3 maximum log reduction in 70 J/cm2 after 75 minutes illumination.

**Figure1:**
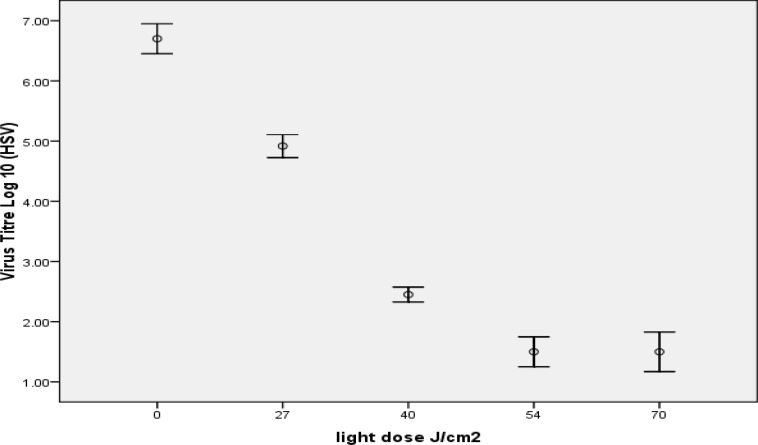
Inactivation of HSV by 1 µM MB in combination with different light dose (depends on different illumination time), the most reduction obtained in 54 J/cm^2^ and increase in light dose decrease virus titration

**Figure2:**
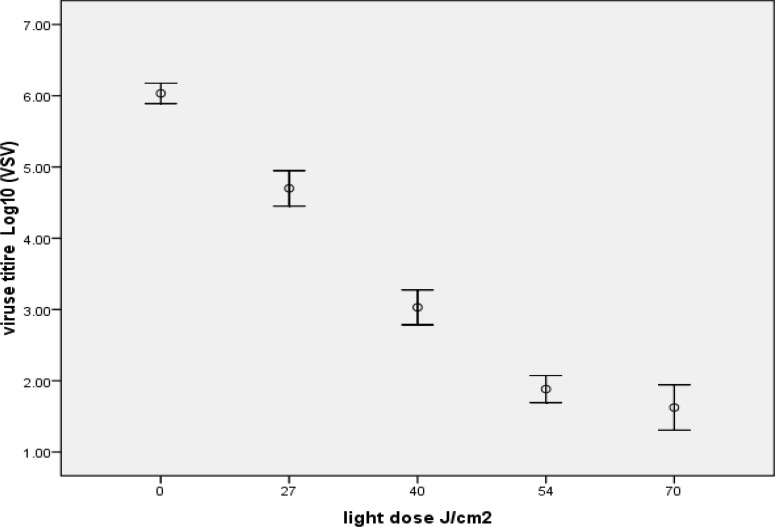
Inactivation of VSV by1 µM MB in combination with different light dose (depends on different illumination time), the most reduction obtained in 70 J/cm^2^ and increase in light dose decrease virus titration

In 1 µm concentration of MB, HSV had 5.20± 0.3 maximum log reductions that obtained in 54 J/cm2 after 60 minutes illumination (Figure 1), and VSV had 4.90± 0.2 maximum log reduction in 70 J/cm2 after 75 minutes illumination (Figure 2).

**Table I:** Inactivation of HSV and VSV in human plasma by combination of 10 µM MB and visible light. Significant difference with initial load(P-value< 0.05).

**Illumination Time**	**Light Dose**	**Log TCID **_50 _ **Viral Titer (mean ** **±** ** SD)(n=3)**		**Log Reduction factor**	
		**HSV**	**VSV**	**HSV**	**VSV**
**-**	Initial Load	6.70±0.10	6.20±0.03		
**0 min**	0 J/cm2	6.50±0.12	6.00±0.05	0.20	0.20
**30 min**	27 J/cm2	<3.10	<3.20	4.60	3.00
**45 min**	40 J/cm2	<1.15	<1.5	5.55	4.70
**60 min**	54 J/cm2	<0.5	<0.7	6.20	5.50
**75 min**	70 J/cm2	<0.5	<0.5	6.20	5.30

**Table II:** Inactivation of HSV, VSV in human plasma by combination of 1 µM MB and visible light. Significant Difference with initial load (P-value < 0.05).

**Illumination Time**	**Light Dose**	**Log TCID **_50 _ **Viral Titer (mean ** **±** ** SD)(n=3)**		**Log Reduction factor **	
		HSV	VSV	HSV	VSV
**-**	Initial Load	6.70±0.10	6.20±0.03		
**0min**	0 J/cm2	6.50±0.12	6.00±0.05	0.20	0.20
**30 min**	27 J/cm2	<4.90	<4.70	1.80	1.50
**45 min**	40 J/cm2	<2.50	<3.00	4.20	3.20
**60 min**	54 J/cm2	<1.50	<1.90	5.20	4.30
**75 min**	70 J/cm2	<1.50	<1.30	5.20	4.90

## Discussion

To ensure the safety of plasma and its derived products, PRT are used in many countries. The major pathogen reduction or elimination methods currently available for plasma involve the use of SD, MB, Amatosalen and Riboflavin as additives.

Many countries interested in MB PRTs to decrease the risk factors in plasma and its transfusion components (10). Photosensitization reactions induced by MB excitation are known to cause damage to viral nucleic acids (21), so it could inactivate transmissible viruses in human plasma. Several studies showed MB treated plasma has no side effect on plasma recipient s health (22, 23). Because of this reason many blood transfusion organizations are using this method for pathogen reduction purposes.

MB based PRTs have been developed in different manner using different dose of MB and light source and illumination time. 

Two parameters that are different in MB based PRTs are light dose and MB concentration. Light dose depends on illumination time and light source. Lambrecht used MB and illumination used halogen to inactivate viruses in plasma for the first time and showed, this technology could decrease HSV and VSV titer more than > 5.0 Log_10_ (21).

In commercial form, the Spring® technology which developed in Spain was the pioneer method. It used fluorescent lamps to activate MB whilst illumination time was 60 minutes, that decrease HSV and VSV titers > 4.9 and > 3.0 Log_10_, respectively (8, 14). The most new version of MB PRT is Theraflex (Macopharma, France), using LED with about 20 minutes illumination time this technology could decrease > 5.5 and > 5.0 Log_10, _of HSV and VSV titers, respectively (21-24).

There are many model viruses that can be used to find if these methods have enough ability for virus inactivation. Using HSV and VSV as models could approve MB ability to inactivate both RNA and DNA viruses. 

Theraflex methodsP- value> 0.05). It is shown that this device could inactivate viruses according to WHO recommendation. Development and optimization reduction in virus titer and decrease time of illumination needs further study. Using red LED lamps was the most advantage in this device. Red LEDs could decrease illumination times because of their power, and it led to cost benefits. Moreover, LEDs, in comparison with other light sources, decrease temperature in illumination period that help to maintain plasma proteins activity. 
